# Social Network Research contribution to evaluating process in a feasibility study of a peer-led and school-based sexual health intervention

**DOI:** 10.1038/s41598-021-90852-w

**Published:** 2021-06-10

**Authors:** Chiara Broccatelli, Peng Wang, Lisa McDaid, Mark McCann, Sharon Anne Simpson, Lawrie Elliott, Laurence Moore, Kirstin Mitchell

**Affiliations:** 1 Institute for Social Science Research, The University of Queensland, Brisbane, Australia; 2 Centre for Transformative Innovation, Swinburne University of Technology, Melbourne, Australia; 3 MRC/CSO Social and Public Health Sciences Unit, University of Glasgow, Glasgow, UK; 4 Department of Nursing and Community Health, Glasgow Caledonian University, Glasgow, UK

**Keywords:** Clinical trial design, Randomized controlled trials, Medical research, Epidemiology

## Abstract

There is growing interest in social network-based programmes to improve health, but rigorous methods using Social Network research to evaluate the process of these interventions is less well developed. Using data from the “STis And Sexual Health” (STASH) feasibility trial of a school-based, peer-led intervention on sexual health prevention, we illustrate how network data analysis results can address key components of process evaluations for complex interventions—*implementation*, *mechanisms of impacts*, and *context.* STASH trained students as Peer Supporters (PS) to diffuse sexual health messages though face-to-face interactions and online Facebook (FB) groups. We applied a Multilevel Exponential Random Graph modelling approach to analyse the interdependence between offline friendship relationships and online FB ties and how these different relationships align. Our results suggest that the creation of online FB communities mirrored offline adolescent groups, demonstrating fidelity of intervention delivery. Data on informal friendship networks related to student’s individual characteristics (i.e., demographics, sexual health knowledge and adherence to norms, which were included for STASH), contributed to an understanding of the social relational ‘building’ mechanisms that sustain tie-formation. This knowledge could assist the selection of opinion leaders, improving identification of influential peers situated in optimal network positions. This work provides a novel contribution to understanding how to integrate network research with the process evaluation of a network intervention.

## Introduction

There is growing interest in the use of network interventions to improve Public Health. Rather than focusing on individuals, network interventions try to change people’s behaviour through proximal social contacts and diffusion of healthy behaviours by persuasion, information flow, modelling behaviours, and social influence^1,2^. Network interventions vary in their focus and design. Most commonly, they can (a) aim to reach marginalised people, (b) accelerate information cascade through peer-to-peer interactions, (c) favour new relationship opportunities, or (d) use opinion leaders to promote behaviour change^3,4^. Network interventions that identify influential individuals to act as opinion leaders (category d), also called *peer-led* interventions, are based on the premise that individuals are susceptible to peer influence and adopt behaviours in order to fit in with their friends^5^. Peer-led interventions have shown some potential, often providing better outcomes than individual-level approaches^2,6^. This is evident across a range of health issues including reducing smoking behaviours^7^, drug misuse and promoting condom use^8–11^.

Since the key component of a peer-led intervention is the process of social influence activated by people’s social networks, one way to design, implement, and evaluate this type of intervention is to apply network methods. These can provide the framework and data analysis tools to facilitate mapping, measurement, and understanding of influence behaviours and, more generally, social relationships^12,13^. Network methods can control for the interdependencies of social ties, enabling one to adjust for the fact that the presence of a tie is conditional on the presence (or absence) of other ties. In contrast, traditional analyses of variance and regression models simply assume independence of observations^14^. Consider, for example, friendship formation. Assuming the dependence of observations means the fact that one nominates the other as a friend may be dependent on being nominated by the same other person as a form of reciprocity. Assuming the independence of network tie observations, instead, implies that the simple propensity for reciprocity does not exist, and friendship just happens by chance.

Social network methods have been used in several ways to develop and understand Public Health interventions. They have been used to map complex systems in intervention development by, for example, mapping the relationships between research staff, policy makers and stakeholders^15–17^. They have also been applied to capture the occurrence of behaviour changes due to an intervention and identify which, and how, social relationships successfully impact or mediate the intervention effects^18–21^. While the focus to date has primarily been on explicating mechanisms of change within interventions, there are other aspects than need to be considered. The UK Medical Research Council (MRC) guidance on process evaluation^22^ highlights the importance of: (1) implementation (whether the intervention was conducted and delivered as planned and with fidelity), (2) mechanisms of impact (exploring the mechanisms though which the intervention brings about change), and (3) context (investigating factors which influence the intervention implementation and its effects). Here we make a novel contribution by showing how network research can support understanding of process evaluation—implementation, mechanisms of impact, and context—of an intervention, including the perspective of social relationships. Specifically, we show how a particular class of network statistical models—Multilevel Exponential Random Graph Models (MERGMs), can address process questions in interventions engaging multi-level networks. MERGMs are novel methodological tools that allow statistical inference using network data so that researchers can study cross-level interdependent social processes and mechanisms.

## Methods

### STASH intervention

The intervention we refer to in the present work is “STis And Sexual Health” (henceforth STASH); a feasibility trial of a school-based, peer-led, sexual health intervention. We use network data collected as part of the larger evaluation study to illustrate our approach. A full description of the trial has been published previously^23^ and the trial results are available here^24^. STASH was adapted from an effective, informal school-based peer-led intervention for smoking prevention in adolescence (ASSIST)^7^. Diffusion of innovation theory^25,26^ - in which opinion leaders spread information designed to change attitudes and beliefs and encourage behaviour change - was the theoretical framework for both studies. The STASH trial tested the feasibility of using peer-supporters (henceforth PS) to intervene in order to enhance STI-prevention and promote sexual health through online networks and face-to-face interactions among students (Scottish S4, aged 14–16 years) in six secondary schools in Scotland (UK). As feasibility study, STASH was designed to test acceptability, fidelity and reach of the trial, and to establish if the program met the progression criteria to proceed further with a full evaluation. That being the case, the focus of the study was on process and not outcome.

PS were identified through peer-nominations by all S4 students, then recruited, trained and supported to deliver the intervention to their S4 peers. After two-days of training, PS were asked to interact face-to-face with their peers as well as use their existing Facebook accounts to set up closed FB groups and invite their friends to join these. FB closed groups were used because of the sensitivity of the content, and the need to ensure that PS felt comfortable and in control of who could view it. Here they could share educational material (memes, infographics, quizzes) from a STASH website that housed a curated set of shareable contents related to sexual health. FB groups were monitored by STASH trainers who also met with the PS weekly or fortnightly throughout the period in which they were active (5–10 weeks). Table S1 in the appendix summarises the information related to the STASH intervention. The STASH trial included a comprehensive process evaluation^24,27^. See supplementary material further trial information.

### STASH participants

In STASH students who became PS were nominated by their peers (via an anonymous nomination form) and recruited to the intervention. All students were asked to list up to five individuals in the same year group considered as the most respectful and trustworthy, leaders in sports or other group activities as well as role models and the most persuasive students (the list of different questions asked in each school is reported in the supplementary material, Table S2). A full description of the recruitment process can be found elsewhere^23,24^. The top 25% of S4 students in each school year group receiving the most nominations were invited to a recruitment meeting and on average, 52% of nominated students opted to become Peer Supporters (13% across the entire year group).

As part of the feasibility evaluation, all students in the intervention year were asked to complete a baseline and follow-up questionnaire. This included questions relative to their friendship relationships. Thus, for the network analysis, the sample consisted of 739 students from S4 (age 14–16) in six intervention schools who completed the follow-up questionnaire in June 2018. Table S3 in the supplementary material reports the school level sample sizes. Overall, 79% (response rate) completed the follow-up questionnaire (School 1–71%, School 2–90%, School 3–80%, School 4–48%, School 5–91%, School 6–94%,), (40.7% males and 56.3% females). Since our focus was on intervention processes, we did not examine control data as these participants were not exposed to the intervention.

### STASH network variables

*Friendship network data* came from the friendship module section of the questionnaire. Participants were asked to select up to six of their closest friends outside and inside their school year group using a drop-down menu populated by the school class list. We focused on student’s close friendships because these are known to be linked to students’ health and well-being as well as behaviour and conformity to peer norms^28,29^. Since students did not necessarily nominate the same others from whom they received a friendship nomination, the networks have directionality. To minimise potential bias deriving from ignoring missing relationships in our networks^30^, we retained non-participants, i.e., students who did not complete the survey but were nominated as friends by other students. Moreover, missing ties (e.g., from non-participants to participants and between non-participants) were treated as exogenous network structural controls, meaning our models included the possibility of a tie between a participant and a non-participant while a tie between a non-participant and a participant was considered impossible.

*FB network data* were derived from the log monitor of FB activities, and were organised in a matrix format with rows representing the respondents, and columns containing the ID of the PS who set up the FB groups. The values intersecting each row and column were represented by 0 or 1, with 1 indicating that the respondent joined that FB group. Connections to STASH FB groups were considered as undirected ties. Friendship networks (among node sets A) and STASH FB networks (between node set A and node sets B) were our network variables. Friendship ties were the dependent variables, while FB ties were treated as predictor variables in the MERGMs, in order to test how the online network related to offline friendship tie-formation. Although other data sources were available (e.g., PS activity monitoring log and project monitoring log), this information was not suitable for network methods and are not discussed here.

### STASH individual covariates

We included participant *gender* as a demographic control variable, by using a binary indicator (1 = male, 0 = female) and a binary variable indicating the students who took the role of *PS* (1 = yes, 0 = no). We controlled for several individual attributes associated with adolescent sexual health since they had potential to impact on friendship tie-formation. These variables, derived from students’ questionnaire responses, could be influenced by the intervention as well as a potential proxy measure of the delivery of intervention messages by word of mouth. These variables were: *sexual health-related knowledge*, *adherence to positive sexual health norms,* and *tendency to talk to friends about sexual health matters*. In the supplementary material, a full description of these variables is reported in Table S4, while Table S3 shows descriptive statistics of the individuals’ covariates for the six schools included in this study.

### Analytical method

Network data were analysed by applying MERGMs. These models are part of the Exponential Random Graph Modes family, and allow statistical inference using network data (one-to-one connections). MERGMs are novel tools that allow researchers to study cross-level interdependent relational mechanisms. These mechanisms explain how the network is built, in the way that one might identify the bricks that compose a wall. If you know how many bricks you have and what their material is, you might imagine how tall and strong your wall is going to be. With these statistical tools, researchers model the observed counts of the local network configurations of ties representing social mechanisms (e.g., reciprocity—I am your friend so you are mine; transitivity—friends of my friends are also my friends; etc.) as graph statistics and compare them with what they could otherwise obtain from a distribution of random graphs (see^14,31,32^). While ERGMs only allow the examination of one network, MERGMs facilitate the examination of two interrelated networks that are considered on different levels, for example understanding how relationships and inter-group ties may affect one another through group affiliations^33,34^. This means considering how ties present at one level (node set A) determine the presence of another level of relationships (node set B), while taking into account that some network processes are acting within each level. A detailed description of MERGMs and their application in the context of a community-based systems intervention was recently published^17^, and we build on this in the work reported here.

**Figure 1:**
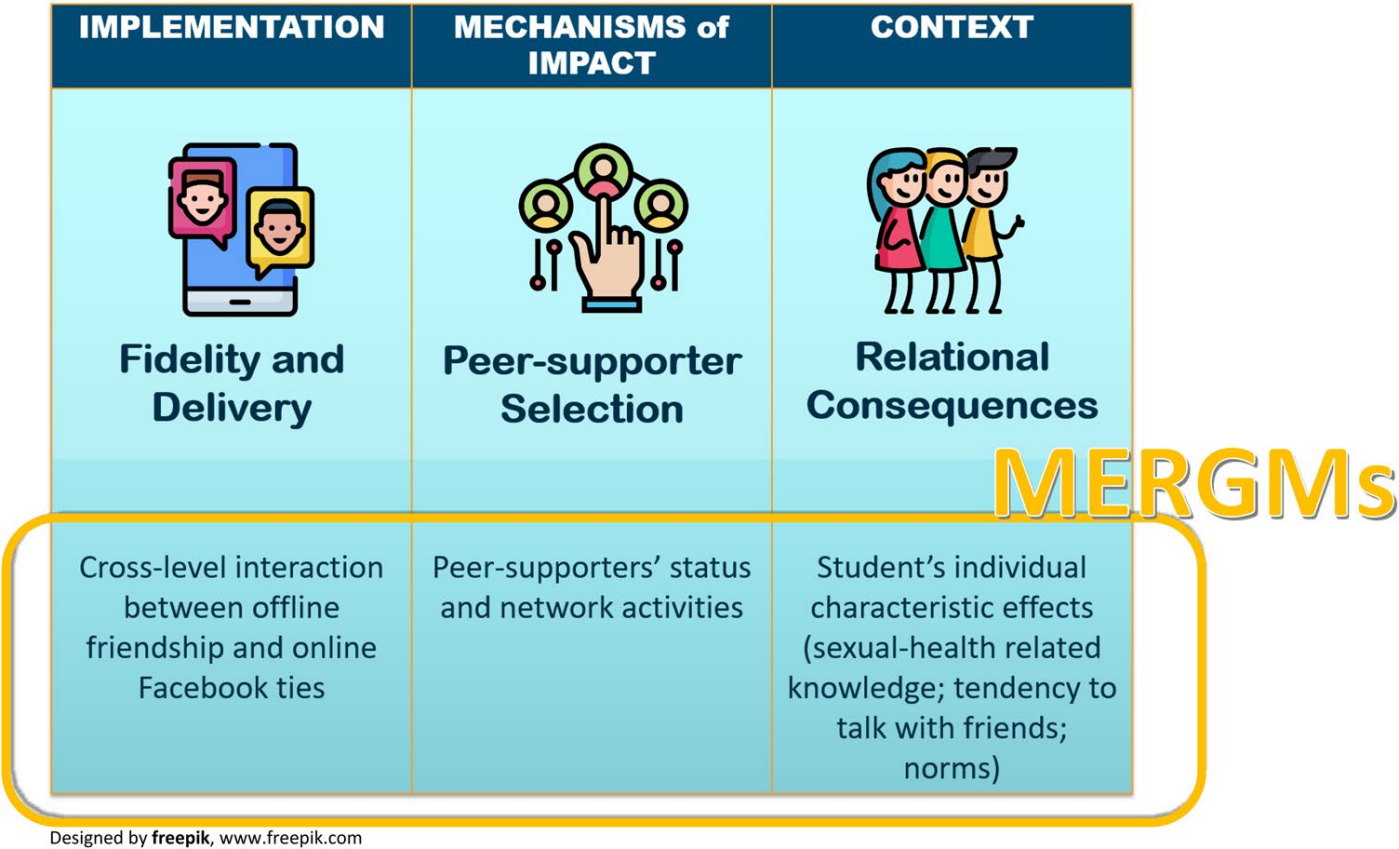
Social Network results and process evaluation.

We ran a MERGM separately for the six intervention schools in order to highlight any different network processes in each school. One school was very small and classes in the senior years (S4, S5, S6), were mixed, yet only S4 students were surveyed. We omitted these from the results and discussion but include them in the supplementary material (Table S5). Our variables of interest comprised the offline friendship ties among students (node set A) and student’s embeddedness in online FB groups (node set B) and we expected this to be associated with the distribution of offline friendship ties. Offline and online ties were theorised in a cross-level design because students could be members of several FB groups and because the presence of a friendship tie could determine the affiliation of a student in a FB group. The effects of young people’s knowledge about sexual health, their attitudes, and their tendency to talk about sex and sexual health with friends, were included as continuous variables, and were considered as predictors; participant gender and PS’ status were included as control variables. Figure 1 summarises how network generated results from the MERGMs contributed to understanding the three elements of the process evaluation.

In this paper we sought to use MERGMs to explore: intervention fidelity (whether or not the PS acted according to the planned activities and how students responded); potential mechanisms of change relating to the potential of PS to deliver content (were PS well posited in their social networks and able to reach their friends?); and the social context in which the intervention was delivered (were there any unintended consequences for friendships that may have affected the delivery of the intervention due to, for example, the sensitivity of the topic?). These questions were addressed in the STASH feasibility trial^24^ and here we examine how MERGMs can add to this understanding. In relational terms, the social context of the intervention consists of the pattern of friendships among students and the cross-level presence of friendship ties and FB ties (it is worth noting here that the wider social context of the STASH intervention also included relationships between school staff, teachers, students and peer supporters but social network data is only available for students). The STASH intervention created new online groups that capitalised on existing overlap between offline and online friendship connections within a school year group^35^. By exploring the relational aspects of these processes with MERGMs we aimed at understanding: how the presence of friendship ties between students at one level (node set A) is associated with the presence of affiliation ties on FB groups at another level (node set B). In this way, we sought to assess whether the creation of online communities on FB mirrored the offline adolescent groups that we expected to be aligned as per STASH instructions and delivery, and provide an understanding of the use of FB social media in disseminating prevention messages on sexual health among friends^36^. In order to evaluate the potential mechanisms of impact and the contextual processes related to the STASH intervention, we focused on how student-to-student relationships were shaped and formed, taking into account students’ characteristics and the role of PS. Finally, we explored how adherence to norms, sexual health knowledge as well as being, or not being, a PS could explain the presence of friendship nominations.

### Model specification

Our goal was to examine how FB ties and individuals’ characteristics (i.e. gender, being a PS, knowledge, adherence to norms and tendency to talk with friends) was associated with friendship tie-formation. Prior research on class friendship networks has shown that they are made by self-organising processes following which an occurring tie between two individuals can be dependent on the relationships they have with others, beyond the dyad^31^. In particular, strong ties such as close friendship relationships tend to be intimate, reciprocal and clustered, where friends of friends are more likely to become friends^37^, and especially among females and in small networks^38^. Students have a strong tendency to form same-gender friendship ties^39^, and influence each other’s behaviour both offline^40^ and online^41^. In order to assess if these tendencies were present in the sample networks, we used several parameter effects.

**Figure 2:**
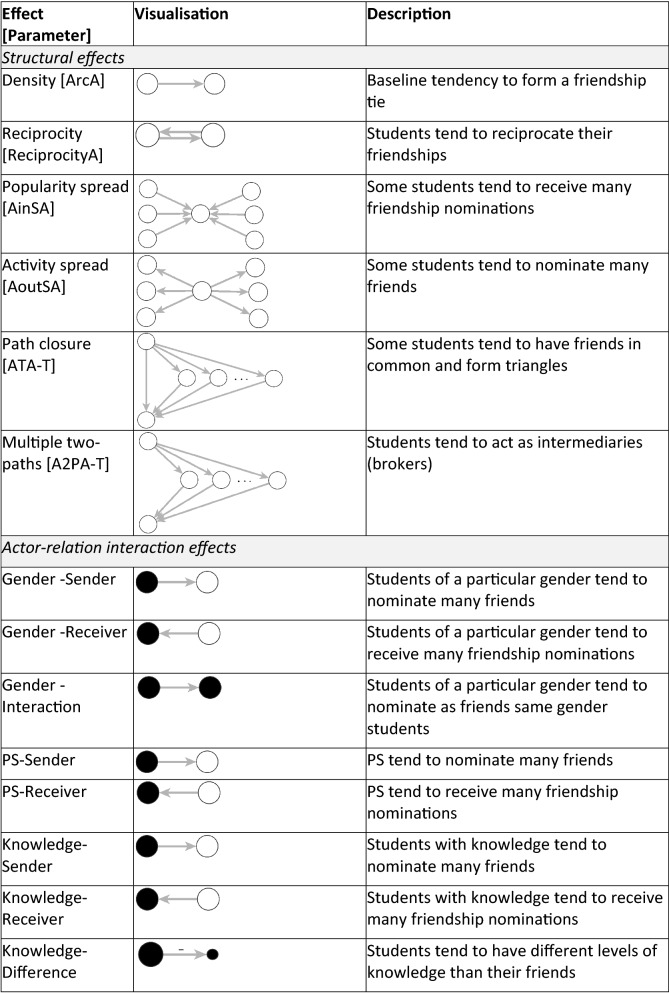

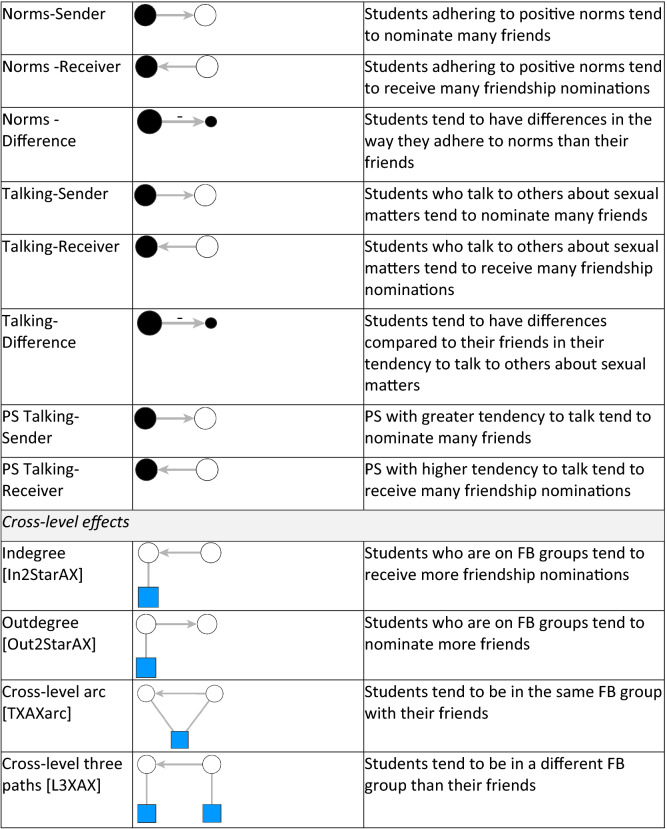
Parameter effects, visualisation and description.

We used the freely available software MPNet for all estimations^42^. The effects specified in our models (with MPNet parameter name in square brackets) are listed and described in Fig. 2. First, we included *structural effects* such as the tendency to form a friendship tie [ArcA] and to reciprocate [ReciprocityA]. We also controlled for network degree distributions for both in-going and out-going friendship ties (popularity spread [AinSA]; and activity spread [AoutSA]). Since students were requested to make up to six friendship nominations, the parameter estimation for Activity spread was conditional on a maximum out-degree of six. Moreover, we included the Path closure parameter [ATA-T] to control for the network tendency toward closure which can be revealed by the presence of friendship triangulations (being a friend of friend) and suggests the students’ tendency to cluster together in small groups. Finally, we also controlled for the network tendency of brokerage, represented by the Multiple two-paths parameter [A2PA-T], that in our case, corresponds to the situation where students act as intermediaries between two other students that are not directly related.

The second part of the model includes parameters known as *actor-relation interaction effects.* For each variable corresponding to individual covariates (gender, knowledge, norms and tendency to talk), we used three basic interaction effects: sender, receiver and interaction or difference. These effects control for the main impact of participant’s characteristics on tie-formation. The sender effect concerns the tendency for individuals with the given attribute to nominate many friends (being active); the receiver effect refers to the tendency for individuals with the given attribute to receive many friendship nominations (being popular). The interaction effect represents homophily, that is the tendency for individuals with the same attribute (categorical in our case) to be friends. The difference effect, specifically for continuous attributes, refers to the heterophily effect, the absolute difference in score of a given attribute among two individuals. Both interaction and difference effects control for homophily, that is the tendency for individuals to befriend alters that are similar to them.

The third part of the model controls for the interaction effects between the two types of networks, offline friendship and online FB group affiliation. Indegree parameter [In2StarAX] refers to the tendency for students who joined a FB group to be popular and receive many friendship nominations. Outdegree parameter [Out2StarAX] looks at the tendency for students who joined a FB group to be active and nominate many other as friends. Affiliation based Cross-level closure parameter [TXAXarc] controls for the tendency for students who are friends offline to affiliate with the same FB group, while the Cross-level three paths parameter [L3XAX] shows the tendency for students who are friends to join different FB groups.

Guidance suggests that it is inadvisable to interpret the magnitude of the parameter estimates of MERGMs by calculating their probability because each effect is conditional to all the other ones included in the model, and they should not be interpreted singularly^43^. In addition, parameter estimates for networks of different sizes are not comparable across them. As the effect sizes are applicable only to the specific population being modelled, here our interpretation is only related to each specific school context. Another important consideration is whether the effect estimates resulting from ERGMs are trustworthy. The DELTA^2^ guidance^44^ highlighted concerns about using effect estimates resulting from pilot studies due to the lack of statistical power, however this does not apply in the case of MERGMs. As opposed to conventional statistical approaches, the numbers of observations based on the network size (n), where n(n − 1) numbers of tie variables that are modelled.

MPNet estimates model parameters using Monte Carlo Markov Chain Maximum Likelihood Estimation (MCMCMLE) method^45^, and performs model Goodness of Fit (GOF) test to assess whether the fitted model can adequately represent the various characteristics of the real networks, including graph statistics that are not part of the model. The GOF analyses are reported in Table S6 in the supplementary material. Results show that the fitted models provide adequate fit to the observed network data.

## Results

**Figure 3:**
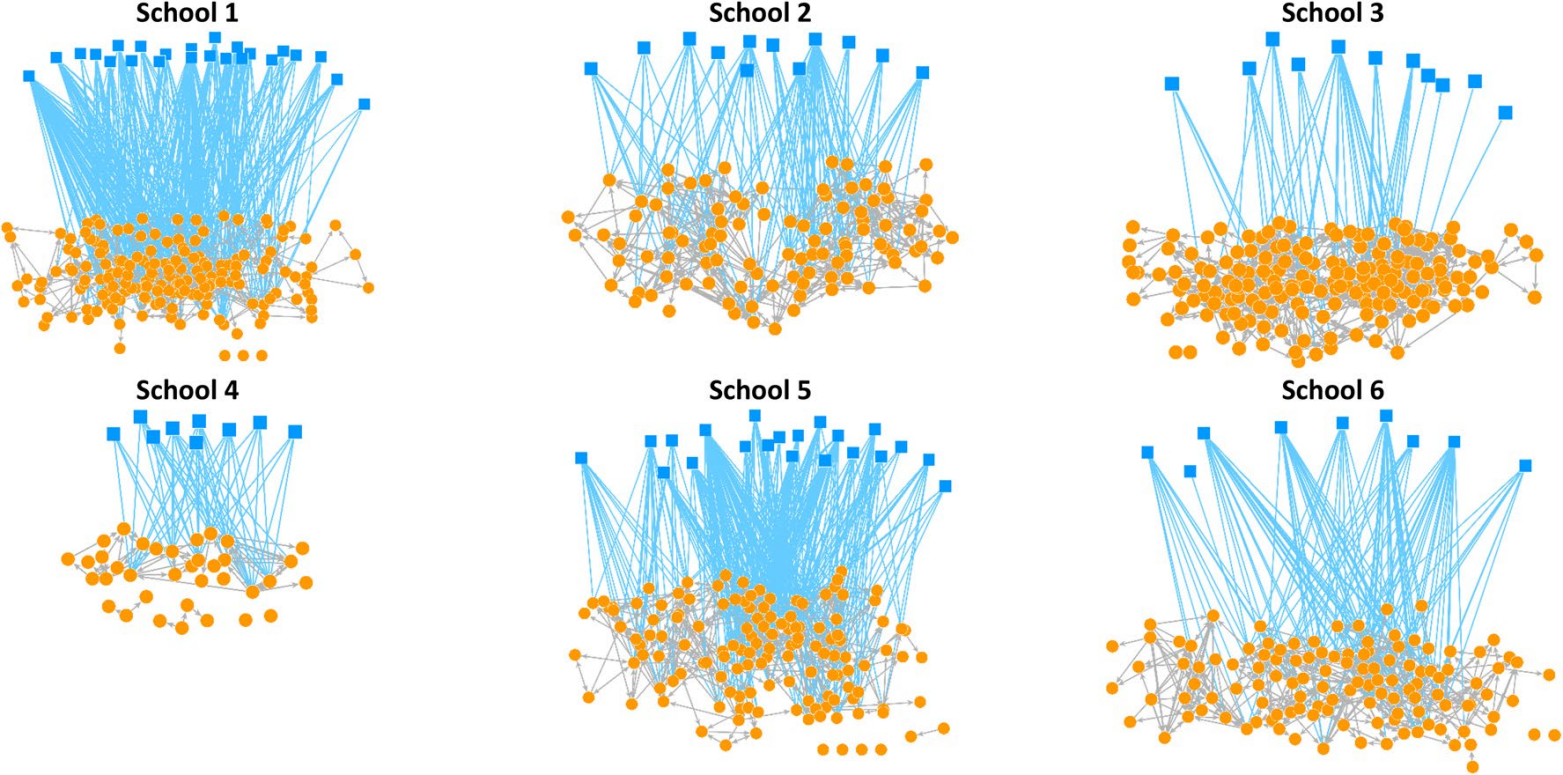
Visualisation of multilevel network data for each school. Students are shown as circles and the friendships among them as a line (grey). FB groups are represented in squares and a tie between circles and squares indicates student’ affiliation to FB groups. Students can have multiple FB memberships.

In the visual representation, students are depicted as circles and the friendships among them as a line, where FB groups are squares and ties between circles and squares indicate students’ affiliation to FB groups (Fig. 3.).

The parameter estimates (and estimated standard errors) for the fitted MERGMs are shown in Table 1. We denoted a parameter significant when its ratio to its estimated standard error is greater than 2. Significant parameters are designated by an asterisk (*). A significant and positive parameter suggests that the corresponding configuration takes place more in the observed network than would be expected by chance; a significant and negative parameter suggests, instead, that the configuration is rarer than what would be expected by chance. There are a large number of possible ties even for networks involving not many people. As a result, we can be confident that our effect estimates are adequate and have enough statistical power to detect endogenous and exogenous effects but, of course, only in respect to the schools under examination.

**Table 1:** Parameter estimates for MERGMs for each intervention school. Significant parameters (in bold) and designated by an asterisk (*).

	School1			School2			School3			School5			School6		
Effects	Parameter	Stderr		Parameter	Stderr		Parameter	Stderr		Parameter	Stderr		Parameter	Stderr	
*Structural Effects*															
Density [ArcA]	**− 3.681**	**0.412**	*	**− 3.211**	**0.536**	*	**− 3.855**	**0.382**	*	**− 3.973**	**0.413**	*	**− 2.476**	**0.493**	*
Reciprocity [ReciprocityA]	**3.103**	**0.247**	*	**1.866**	**0.238**	*	**2.705**	**0.207**	*	**2.894**	**0.243**	*	**1.926**	**0.24**	*
Popularity spread [AinSA]	**− 0.74**	**0.17**	*	**− 0.8**	**0.249**	*	**− 0.368**	**0.14**	*	**− 0.47**	**0.179**	*	**− 0.596**	**0.186**	*
Activity spread [AoutSA]	**0.768**	**0.153**	*	**1.057**	**0.208**	*	**0.789**	**0.182**	*	**0.659**	**0.182**	*	**0.364**	**0.216**	
Path closure [ATA-T]	**1.048**	**0.08**	*	**0.975**	**0.08**	*	**1.249**	**0.061**	*	**1.173**	**0.08**	*	**1.292**	**0.079**	*
Multiple 2-paths [A2PA-T]	**− 0.14**	**0.032**	*	**− 0.203**	**0.034**	*	**− 0.145**	**0.023**	*	**− 0.104**	**0.031**	*	**− 0.144**	**0.03**	*
*Actor-relation interaction effects*															
Gender-Sender	**− 0.761**	**0.19**	*	**− 1.806**	**0.418**	*	**− 0.73**	**0.141**	*	**− 0.69**	**0.171**	*	**− 0.474**	**0.166**	*
Gender-Receiver	**− 0.496**	**0.183**	*	**− 1.409**	**0.344**	*	-0.12	0.103		**− 0.419**	**0.167**	*	**− 0.539**	**0.17**	*
Gender-Interaction	**1.35**	**0.234**	*	**3.516**	**0.515**	*	**0.883**	**0.12**	*	**0.942**	**0.191**	*	**1.144**	**0.181**	*
PS-Sender	0.061	0.281		**− 0.927**	**0.364**	*	**1.304**	**0.628**	*	-0.605	0.315		0.158	0.36	
PS-Receiver	0.292	0.316		0.272	0.364		-0.205	0.393		**0.824**	**0.267**	*	0.093	0.283	
Knowledge-Sender	**0.109**	**0.049**	*	0.03	0.06		**− 0.099**	**0.046**	*	0.039	0.051		0.001	0.051	
Knowledge-Receiver	-0.006	0.057		-0.04	0.064		0.041	0.039		0.004	0.054		-0.007	0.044	
Knowledge-Difference	-0.025	0.042		-0.032	0.055		**− 0.091**	**0.025**	*	**− 0.111**	**0.038**	*	**− 0.128**	**0.028**	*
Norms-Sender	-0.061	0.044		0.033	0.049		0.035	0.046		-0.079	0.049		-0.052	0.051	
Norms-Receiver	0.05	0.053		0.049	0.053		-0.001	0.039		**0.107**	**0.05**	*	-0.017	0.043	
Norms-Difference	**− 0.104**	**0.043**	*	-0.065	0.044		**− 0.088**	**0.029**	*	-0.061	0.04		-0.008	0.04	
Talking-Sender	0.012	0.035		**− 0.098**	**0.046**	*	0.049	0.037		**− 0.134**	**0.045**	*	-0.025	0.041	
Talking-Receiver	-0.058	0.046		**0.223**	**0.049**	*	-0.02	0.029		**0.151**	**0.047**	*	**0.072**	**0.036**	*
Talking-Difference	**− 0.069**	**0.028**	*	-0.035	0.034		**− 0.073**	**0.02**	*	**− 0.071**	**0.028**	*	**− 0.07**	**0.028**	*
PS Talking-Sender	0.031	0.086		**0.635**	**0.225**	*	**− 0.424**	**0.173**	*	0.118	0.103		-0.086	0.16	
PS Talking-Receiver	0.026	0.087		**− 0.367**	**0.176**	*	0.134	0.113		-0.046	0.074		0.136	0.105	
*Cross-level effects*															
Indegree [In2StarAX]	0.01	0.037		**0.129**	**0.063**	*	-0.009	0.068		**− 0.077**	**0.037**	*	-0.017	0.042	
Outdegree [Out2StarAX]	-0.028	0.032		**− 0.204**	**0.07**	*	0.013	0.096		0.045	0.038		-0.086	0.05	
Cross-level arc [TXAXarc]	**0.415**	**0.048**	*	**0.627**	**0.117**	*	**0.793**	**0.243**	*	**0.294**	**0.041**	*	**0.535**	**0.107**	*
Cross-level 3-paths [L3XAX]	**− 0.024**	**0.006**	*	**− 0.052**	**0.018**	*	-0.117	0.076		**− 0.02**	**0.005**	*	**− 0.049**	**0.022**	*

We present our results starting with the structural effects, and then refer to the key components of process evaluation as reported in the MRC process evaluation guidance.

### Structural effects

Structural characteristics of friendship ties among students were similar in all schools. Students reciprocated their friendships (positive ReciprocityA) and formed cohesive subgroups where friends of friends tended to also be friends (positive ATA-T). Situations where students acted as intermediaries were less likely (negative A2PA-T). Only a few participants were popular and received a high number of friendship nominations (negative AinSA); the majority of students tended to list similar numbers of a few friends (positive AoutSA).

### Cross-level effects linked with implementation fidelity and delivery

Friendship ties (node set A) were aligned with online FB group ties (node set B) in all intervention schools. Students who were friends offline were more likely to join the same online STASH FB groups (positive TXAXarc) confirming, as expected, that the creation of online FB communities mirrored offline adolescent friendship groups. This result speaks to the implementation fidelity of the intervention because it provides the statistical evidence that PS acted as per STASH instructions (they did invite their friends to join closed FB groups) and their student friends responded positively to this invitation. Additionally, results related to tie-formation mechanisms among offline and online ties highlighted that there was no clear pattern across schools in terms of student popularity (popularity spread—indegree), student activity (activity spread—outdegree) and FB membership. Only in school 2, for example, students who joined STASH FB groups were more popular (positive In2StarAX), but nominated fewer friends than other students (negative Out2StarAX).

### Actor-relation interaction effects linked with mechanisms of impact

Analysis by PS position within their social networks revealed a mixed picture about their capacity to send or receive more friendship nominations than other students. PS nominated about the same number of friends as other students, except in school 2, where PS nominated less friends (negative PS-Sender) and in school 3, where, instead, PS nominated more friends (positive PS-Sender). Only in school 5 were PS popular and received many friendship nominations (positive PS-Receiver). Only in school 2 did PS with greater tendency to talk about sexual matters with friends nominate more friends (positive PS Talking—Sender) but they were also less popular than other students (negative PS Talking—Receiver). These rather mixed results indicate variation across schools in PS popularity and ability to reach friends based on PS network position and their willingness to talk to their friends about sexual matters.

### Actor-relation interaction effects linked with context

These results highlight some specific properties of the relational context in which the intervention took place. Friendship tie-formation seems to be partially explained by students’ individual characteristics such as student’s knowledge about sexual health, their adherence to positive norms, and their tendency to talk about sexual matters with friends. The homophily effect was significant: in the majority of schools (school 1, 3, 5 and 6) students with a similar tendency to talk (i.e. a lower ‘difference in talking’ variable score between the two students) were more likely to be friends (negative Talking-Difference). Students were selective in their friendship nominations also in respect to knowledge (school 3, 5 and 6): they tended to list as friends the individuals with similar knowledge (negative Knowledge-Difference). Furthermore, in school 2 and 5 students with the tendency to talk about sexual matters were more likely to be considered as friends than others (positive Talking-Receiver), but they did not give many friendship nominations (negative Talking-Sender) either. With respect to positive norms, in most schools, students adhering to positive sexual norms were neither more nor less popular than others. The exception was school 5, in which students adhering to positive norms were more popular among their friends (positive Norms-Receiver). Overall, differences in norm adherence did not affect friendship nominations, but in school 1 and 3, students adhering to positive norms were less likely to be considered friends by others adhering less to positive norms (negative Norms-Difference), indicating homophily. Relative to gender, young men nominated fewer friends (negative Gender-Sender) and in four schools young men received less friendship nominations than young women (negative Gender-Receiver). Finally, students were more likely to nominate same-gender peers as friends (positive Gender-Interaction).

## Discussion

As interest in network research in public health has increased, recognition of the benefits of applying them to understand the relational processes that come into place in social network interventions has gained ground. We found that overall students’ relationships tended to be reciprocal and with closure (e.g., path closure). In relation to cross-level effects, it emerged that STASH FB groups were more likely to connect students who were already friends offline, confirming that offline-online relationships overlap and are tied together^46^. Concerning actor-relation effects of visible attributes, the differences in parameter significance across the networks indicated that actor characteristics played out in different ways in each school. Overall, friendship selection was gender specific, but some students befriended others most similar to them either in terms of tendency to talk about sexual matters, or in respect of sexual health knowledge, or in relation to adherence to positive sexual norms. As suggested in McPherson and colleagues'^39^ influential work on homophily and heterophily, individuals’ similarity is a very important mechanism that explains connections. Both the results from cross-level and actor-relation effects suggest that MERGMs results may be important in explaining tie-formation mechanisms with a view to inform process evaluation.

Previous examples of network research and methods applied for interventions include investigations of social influence on norms and behaviours. Network research can be applied to predict how individuals’ behaviours are influenced by their friends or others to whom they are closely tied (e.g., friends, family members, or more generally one’s intimate relationships). This research has shown, for example, that adolescents who smoke tend to cluster with friends who also smoke, and this tends to incentivise smoking behaviour^19^. This highlights the importance of developing programs that motivate adolescents to befriend non-smokers, rather than just focusing on changing individual’s smoking behaviour^19,20^, in order to prevent the formation of school clusters that reinforce deviant behaviours^21^. By applying network statistical models in this paper, we have contributed to work on the utility of these techniques in understanding network social processes. We have shown how MERGMs can be useful for uncovering process in interventions engaging multi-level networks. While the aforementioned literature mostly focuses on understanding mechanisms of change, ours is among the first to broaden and link this understanding to wider elements of a process evaluation—*implementation*, *mechanisms of impacts*, and *context*. In particular, interdependent social processes (e.g., interpersonal friendship relationships and FB group affiliations) represented as cross-level effects within the MERGMs framework provided evidence of the delivery of intervention activities and their fidelity. Moreover, applying these statistical models offered an understanding of potential relational factors that explained tie-formation and the selection of friends’ mechanism, and gave insights into PS ability to spread the intervention content considering their network position.

It is important to note that statistical modelling techniques bring better insights compared to only using network measures that analyse network topology characteristics, such as centrality, cohesion, clustering. Robins and colleagues^31^ summarised the potential of network modelling techniques indicating they: (1) better capture the social processes coming into play while taking into account the uncertainty of them happening; (2) explain the actual mechanisms that led to a certain network property since different processes can result in a similar network characteristic (e.g., network clustering might be due to the presence of (endogenous) structural balance or (exogenous) homophily effect); and (3) understand the distribution of social processes, where exactly they are occurring within the network. Social processes, in fact, could only characterise specific network areas rather than the whole network homogeneously. This explains why some of our findings differ from the ones presented in the main STASH trial analyses, which are based on network descriptives^24^. In the following discussion, MERGM findings are summarised by referring to key process evaluation components (as theorised in Fig. 1) and the current literature in the field of public health and network research.

### Implementation fidelity and delivery: providing evidence that the PS acted according to the planned activities and delivered the intervention with fidelity

FB relationships were more likely to be present if sustained by offline relationships; in line with the network theoretical assumption that relationships are interdependent. The formation of FB communities mirrored offline adolescent groups, thus confirming fidelity of intervention delivery, since PS were asked to set up FB groups and invite their friends. Our findings also reinforce the potential utility of retrospectively exploring peer networks and the formation of online relationships to explain the acceptability of suggested behaviours. For example, we might suggest that overall, joining a Facebook group was a fairly accepted behaviour and students were receptive to the idea. Other findings of the STASH process evaluation reported elsewhere^36^, also confirm different levels of engagement in STASH FB groups in different schools (e.g., school 3 presented the highest prevalence of face-to-face interactions because FB was a less popular platform among their school year group) and that their online engagement in STASH was mostly in parallel with their habitual use of social media. These results highlighted the challenge of engaging youth in social media interventions in sexual health. Literature elsewhere however suggests that social media can be a useful resource for delivering public health services and interventions more generally^47^, creating the environment to diffuse information^48^, and increasing sociopolitical awareness^41^ and civic engagement^49^. Finally, different cross-level effects would have emerged if alternative instructions were given to PS (e.g., asking them to invite not only friends but also friends of friends and acquaintances, or even peers they did not directly know). In this study, friendship relationships and online membership through FB groups assumed a central importance within the design of the intervention. To assess implementation and specific aspects of the intervention design such as fidelity, researchers can collect social network data to assess the extent to which individuals engage with the intervention. Here the key question is: are there any specific types of relationships (e.g., friendships and online membership) among the target population that can help determine if the individuals adopt behaviours being promoted by intervention agents?

### Mechanisms of impact: reflecting on the PS role and their potential to deliver the intervention content

Network results can be used to reflect upon the ability of PS to deliver the intervention content and their potential impact in reaching their friends. MERGMs allowed us to assess if PS were mentioning, and were mentioned by, many friends based upon their role and if their tendency to talk about sexual health related topics with friends was related to the formation of friendship ties. For comparison, STASH main paper findings resulting from the application of network centrality measures for PS across schools reported that, overall, PS received more friendship nominations than other students. With MERGMs we can limit this result, suggesting that a statistically significant higher number of contacts for PS only appeared in two schools (i.e., school 3 for PS nominating friends and school 5 for PS receiving friendship nominations). Thus, we might suggest that PS may only have had a partial ability to reach out to many peers compared to other students because they did not nominate friends nor were they mentioned by many others as friends. This is an important result because it seems to suggest that students’ influential reputation and prestige (as determined by their nomination as PS) did not necessarily overlap completely with students’ popularity among their friends (as determined by their friendship nominations in the friendship module section of the questionnaire) and that students considered as influential leaders were not necessarily the students with more friends. This outcome is consistent with other findings related to peer dominance and health risks behaviours^50^ and indicates that the selection of PS based upon student’s reputation (e.g., considering students that are respected and admired) alone might not be the optimal choice for diffusing sexual health information though friendship networks. Additionally, leaders might not be a suitable choice to inspire changes because they may choose to refrain from leading relational changes that could diminish their leadership status^4^. A possible way to identify influential individuals that can lead changes in behaviour is to include network centrality measures. Centrally located individuals might have a greater potential to diffuse information through their social networks more rapidly than individuals identified using other criteria^4,51^. Other research on opinion leaders applying a network approach has emphasised the importance of selecting opinion leaders who communicate outside their group affiliations (also called boundary spanners), due to their ability to incentivise the cascade of information across different groups and to reach individuals at the margin of local networks^52^. As such, mapping the distribution of informal ties (e.g., collecting network data) before the intervention could inform the selection of trustworthy peer-leaders that are also in optimal structural network positions, with the greatest potential to diffuse information intervention content beyond their immediate contacts. However, how to apply centrality measures in intervention design for peer selection of PS is an aspect that needs to be considered carefully. If we omit the PS nomination exercise, we would lose the endorsement that PS receive through it. This motivates PS to take up the role and complete it and is a key mechanism of change. Its removal could have a negative impact on intervention fidelity. Further research is necessary in this regard. To assess mechanisms of impact, researchers can use social network methods to reflect upon which are the most appropriate actors to be selected as change agents. For example, if an intervention aims at reducing network segmentation, the engagement of individuals that assume brokerage positions might lead to a higher increase of connectivity among disconnected groups within the program recipients. Key questions are: Which individuals are best positioned to spread intervention messages? Could network data be collected prior the identification of leaders? And if so, who are the most appropriate actors to be recruited to act as agents of change based on their network positions?

### Context: identifying other intervening relational factors that influence intervention impact

Exploring the context in which an intervention is delivered is important because pre-existing factors (i.e., considering the roles of different intervention components) as well as different settings may radically impact people’s response to an intervention^53^. This is what the MERGMs do, focusing on how students made friendship nominations considering their friend’s characteristics (i.e., adherence to norms, tendency to talk to friends about sexual matters). This is equivalent to understanding the relational context where person-to-person relationships arise; in other words, the dependence mechanisms that build the networks. Exploring this before implementing an intervention could help to refine its design in order to maximise the diffusion of intervention content. For example, we found that students with a greater tendency to talk about sexual health matters were popular among their friends and mentioned as friends by many and that students with less knowledge were not necessarily gathering information from the most knowledgeable peers, but they tended to exchange information with peers with similar albeit low sexual health knowledge. Work is ongoing to examine these effects with respect to control data for this study. Findings about mechanisms that relate to tie-formation give us important information about the potential unanticipated effects of the intervention, the “collateral effects” that derived from its implementation. For example, one might be interested in knowing if students who were inclined to discuss sexual health topics with their friends were less popular and stigmatised. In our study, students with knowledge on sexual health did not receive fewer friendship nominations than others at follow-up. This information provides reassurance that sharing the intervention content, and possibly the role of being a peer supporter, did not have unintended consequences for their friendship networks.

Additionally, by knowing how the process of tie-formation operates we could think of refining PS recruitment with a view to ensure that they are representative of the different levels of understanding of sexual health topics and propensities in talking about sexual matters. This could potentially maximise student’s learning outcome because we might increase the chances of reaching young people with low baseline sexual health knowledge and low propensity to talk with their friends. However, this cannot be always accommodated. For intervention designs similar to STASH, which include anonymity of the topic ahead of the PS nomination exercise, conducting a baseline survey before or conjointly with the identification of PS might not be feasible. In assessing relational context, social network researchers can contribute understanding of possible negative effects of the intervention. For example, might individuals recruited as PS become less popular as a result of their role? Social network research methods could also assist in considering the broader relational setting, for instance, by gathering information on other level relationships (e.g., student–teacher, teacher-teacher, student-parent, parent-teacher relationships) that could also influence the effectiveness of the intervention.

This study had a number of limitations. We did not assess changes in students’ individual characteristics—PS role, sexual health knowledge, norms and tendency to talk with their friends about sexual-health related matters—at baseline and after the intervention delivery, but we focused, instead, on the second level group structure (FB group membership) that was only available for follow-up data. To control how students aligned their behaviours before and after the intervention, longitudinal statistical modelling techniques for network data are required like stochastic actor-oriented models (SOAM), as demonstrated in previous diffusion studies^54,55^. Future work that applies longitudinal methods for network data will result in a better understanding of mechanisms of change in line with the diffusion of innovation theory. Another avenue worth pursuing is to apply a Bayesian approach to better deal with tie-variable missingness in model-based estimations for network data^56^.

Moreover, future research could consider a wider range of concepts such as individual well-being or individual school performance in order to assess whether other students’ characteristics are related to tie-formation (e.g., exploring whether students who feel isolated and depressed tend to not have many friends in class and through social media). Other behaviours could also be included, for example, whether students that have a tendency to use pornographic material, sexting, or having unprotected sex, affects network choices to become friends. Results from these additional analyses could inform better interventions on sexual health among adolescents. Another limitation is that our findings did not consider the role that adolescences’ sexual identity might play in friendship network formation and Facebook group memberships. Additional large studies are required in this regard. At a methodological level, the network size of friendship social networks might have been closer to reality if friendship choices allowed the nomination of more than six friends for each student. This would result in a more comprehensive representation of friendship networks. Finally, future research exploring cross-level interdependencies between off-line and on-line relationships might benefit from verifying our findings within a different relational intervention context, including by exploring interactions through social networking sites.

Despite the limitations, this work is a first step towards understanding how MERGMs can be helpful in highlighting social processes within multi-level network interventions, as well as contributing to an understanding of intervention process starting from a relational perspective. We demonstrated how network results can be used to improve the assessment of the feasibility of an intervention and can contribute to the process evaluation. We offered meaningful insights on program implementation by suggesting possible implications for the implementation of future network-based interventions. By using network methods that facilitate an understanding of the formation of informal ties, we can go beyond the individual level and provide a more comprehensive investigation of how social networks arise and how relational mechanisms can sustain information diffusion and impact on behaviour.

To better assess the broader relational context, future interventions can also consider the cross-level presence of student-to-student ties in parallel to school staff and teachers’ ties. The MERGMs framework has great potential to explore how these different levels of relationships intersect and provide the basis for knowledge diffusion. Finally, future network interventions might benefit from the analysis of social networks, if possible, conducted before the beginning of the intervention. This can shed light on the existing network processes as well as how the formation of informal ties is regulated by individual and structural network characteristics. This could also potentially offer insights into how the mechanisms of change operate in the given setting and how the social context intersects the diffusion of information. We believe that the use of social network research methods, both prospectively (before implementing the network intervention) and retrospectively (after the network intervention delivery) can be extremely useful and we hope many more researchers will consider this approach.

### Research ethics

The STASH study design was reviewed and approved by University of Glasgow MVLS Ethics committee (ref 200160002). Head teachers agreed to participate on behalf of their schools. Individual written consent was obtained for the peer supporter role and for all students to participate in questionnaires and interviews. All research was performed in accordance with relevant guidelines and regulations.

## Supplementary Information

Supplementary Information.
