# Supplementary material for: Social Network Research contribution to evaluating process in a feasibility study of a peer-led and school-based sexual health intervention
Source: Sci Rep. 2021 Jun 10;11:12244. doi: 10.1038/s41598-021-90852-w (PMC8192897; doi:10.1038/s41598-021-90852-w)
Supplement: Supplementary file 1 — Supplementary Information. [file 41598_2021_90852_MOESM1_ESM.docx]

Social Network Research contribution to evaluating process in a feasibility study of a peer-led and school-based sexual health intervention

Chiara Broccatelli^1^

Peng Wang^2^

Lisa McDaid^1 3^

Mark McCann^3^

Sharon Anne Simpson^3^

Lawrie Elliott^4^

Laurence Moore^3+^

Kirstin Mitchell^3+^

^1^ Institute for Social Science Research, The University of Queensland, Brisbane, Australia.

^2^ Centre for Transformative Innovation, Swinburne University of Technology, Melbourne, Australia.

^3^ MRC/CSO Social and Public Health Sciences Unit, University of Glasgow, UK

^4^ Department of Nursing and Community Health, Glasgow Caledonian University, UK

+ Joint senior authors

Corresponding author: Chiara Broccatelli

Email: c.broccatelli@edu.uq.au

SUPPLEMENTARY MATERIAL

In this section, we present the supplementary material for the readers that complements our publication.

**Table S1. Description of STASH Intervention**

| **The STASH intervention:**  **(1) Peer Nomination.** All students in fourth year of secondary school (aged 14-16) asked to complete a peer nomination questionnaire, comprising 4 questions. Each school used a unique combination of questions; two from the original three questions used in the ASSIST trial (who do you respect; who make good leaders; who do you look up to) and two drawn from four new questions designed for STASH (with whom do you feel comfortable talking about something personal; Whose opinion do you trust; Who is good at persuading others; who is confident at talking to people outside their friendship group). Top 25% of young people receiving most nominations, stratified by gender, invited to recruitment meeting.  **(2) Peer Supporter (PS) Recruitment meeting.**  Trainers introduce STASH and explain the PS role to nominees and address questions. Aim is to recruit 15% of year group.  **(3) Two-day PS training i**n school time, at external venue. PS trained in knowledge, skills, confidence required for role. The training seeks to build motivation, enthusiasm, generate trust, and rapport within PS group and among PS and trainers. PS sign code of conduct during training and agree plan to ‘announce’ the project to year group.  **(4) Peer support work.** (a) PS establish ‘secret’ FB group (invite-only groups; highest privacy setting), comprising friends and STASH trainer. They post messages from the STASH website to this group, and initiate face-to-face conversations centred on STASH messages. They alert friends to the STASH website, and local support sources. PS are supported by a trainer and contact teacher. PS are encouraged to engage with STASH resources flexibly: for instance, in choosing which messages and links to share, and editing messages into their own words if desired. (b) The trainers moderate group discussions, monitor FB posts, support the PS, and facilitate follow-up meetings (weekly or fortnightly) with all PS.  **(5) Acknowledgment of PS efforts.** Certificates, £10 voucher, ‘credit’ toward volunteering award. |
| --- |

**Table S2. Selection of PS: Leadership nomination questions**

| Students were asked to complete an anonymous peer nomination questionnaire. Each school received a different combination of the following questions:  • Who do you respect in S4 at your school?  • Who are good leaders in sports or other group activities in S4 at your school?  • Who do you look up to in S4 at your school?’  • With whom in S4 would you feel comfortable to talk about something personal/sensitive?  • Who in S4 is good at encouraging/persuading others to do things?  • Whose opinion do you trust/value most in S4 at your school?  • Who in S4 is confident at talking to people outside their friendship group?). |
| --- |

**Table S3. Descriptive statistics for students and FB groups in each school**

|  | **School 1** | **School 2** | **School 3** | **School 4** | **School 5** | **School 6** |
| --- | --- | --- | --- | --- | --- | --- |
| ***School networks*** |  |  |  |  |  |  |
| Number of students (n) | 170 | 99 | 176 | 36 | 137 | 121 |
| Number of ties (n) | 424 | 373 | 622 | 53 | 428 | 449 |
| Average friendship ties for students (mean) | 3 | 4 | 4 | 2 | 3 | 4 |
| Students nominating 6 friends (n, %) | 34 (20) | 37 (37.4) | 66 (37.5) | 2 (5.5) | 39 (28.5) | 45 (37.2) |
| ***Actor covariates*** |  |  |  |  |  |  |
| Gender - Male (n, %) | 43 (25.3) | 39 (39.4) | 59 (33.5) | 14 (39) | 43 (31.4) | 49 (40) |
| *Missing (n, %)* | *48 (28.2)* | *8 (8.1)* | *40 (22.7)* | *10 (27.8)* | *27 (19.7)* | *17 (14.1)* |
| Peer-supporters (n) | 23 | 12 | 16 | 7 | 21 | 7 |
| Sexual health-related knowledge (mean, sd) | 1.7 (1.32) | 2.5 (1.1) | 2.2 (1.3) | 1.7 (1.3) | 2 (1.3) | 2 (1.3) |
| *Missing (n, %)* | *51 (30)* | *12 (12.1)* | *41 (23.3)* | *16 (44.4)* | *32 (23.4)* | *15 (12.4)* |
| Sexual health attitudes (mean, sd) | 2.9 (1.4) | 3.1 (1.3) | 3.3 (1.3) | 2.7 (1.3) | 3.2 (1.3) | 3 .1 (1.3) |
| *Missing (n, %)* | *52 (30.6)* | *11 (11.1)* | *37 (21)* | *13 (36.1)* | *32 (23.4)* | *18 (14.9)* |
| Tendency in talking about sex and sexual health (mean, sd) | 2.1 (1.8) | 2.1 (1.6) | 2.1 (1.7) | 2.5 (1.7) | 1.9 (1.7) | 1.7 (1.7) |
| *Missing (n, %)* | *51 (30)* | *14 (14.1)* | *38 (21.6)* | *15 (41.7)* | *27 (19.7)* | *14 (11.6)* |
| ***FB networks*** |  |  |  |  |  |  |
| Number of FB groups (n) | 24 | 12 | 11 | 9 | 21 | 9 |
| Average members for FB groups (n) | 16 | 9 | 5 | 6 | 15 | 14 |

**Table S4. STASH Individual covariate description**

| 1. **Gender** |
| --- |
| Initially, gender was recoded as 1=male, 2=female, 3=other. Nevertheless, only few students selected the other category (18 cases among all respondents (N=739), distributed in four out of six schools). Since numbers within schools were incredibly small to analyse separately as category, we did not retain this information and treated the cases as missing. Thus, for each school included in our models the variable gender presents a binary format (1=male, 0=female). |
| 1. **Sexual health related knowledge** |
| We created an index of *Sexual health-related* *knowledge* ranging between 0-4 derived from number of correct answers to the questions: “Do you think the following statements are true or false (True, False, Don’t know)? 1. With a condom on, a guy should wait until the penis is soft before pulling out after ejaculation; 2. If you get chlamydia you will know because you will have symptoms; 3. If someone under 16 goes to the doctor for an STI test or pregnancy test, the doctor has to tell their parents; 4. A girl can't get pregnant if the guy pulls out (withdraws) before ejaculation/cumming”. |
| 1. **Adherence to positive sexual health norms** |
| We also created an index of *Adherence to positive sexual health norms*, ranging from 0-5 based on a positive response (given in brackets) to four statements deriving from the questions: “Below are some things people say about relationships and sex (oral, vaginal or anal) (Strongly agree, Agree, Neither agree nor disagree, Disagree, Strongly disagree). 1. It is OK for someone who is not your boyfriend/girlfriend to ask you to send a nude pic of yourself (disagree). 2. The sex shown in pornography is similar to sex in real life (disagree). 3. Keeping a partner happy is a good reason to agree to sex (disagree). 4. It is NOT ok to have sex with someone who is very drunk (agree). 5. As long as you use a condom some of the times you have sex, your risk of getting a sexually transmitted infection is very low.”   1. **Tendency to talk to friends about sexual matters** |
| *Tendency to talk to friends about sexual matters* refers to the extent to which students engaged in conversations (1= yes; 0= no) on different topics. This was assessed by the question: “In the last four weeks, have you talked to your friends about any of the following: 1. How you feel about your body or looks; 2. What makes a good or bad relationship; 3. Knowing if you are ready to have sex; 4. Sexually transmitted infections; 5. Sexting (sending nudes or sexual texts) or pornography”. We extracted binary covariates for each single item, giving 1 if the answer was ‘yes’ and 0 in the case of ‘no’ or non-response. Items were then combined on an index varying from 0 to 5. |

In the case of missing responses for the three indexes (Sexual health-related knowledge, Adherence to positive sexual health norms, Tendency to talk to friends about sexual matters) as well as gender, a value was imputed by using the predictive matching mean (PMM) procedure as a single imputation method in order to replace the missing information with the most plausible value starting from the distribution of completed observations^1,2^. While a multiple imputation procedure following Rubin’s method is considered the best approach to deal with missingness in traditional statistics, the complexity of network data structure requires the use of more sophisticated techniques to assess missing information on individual covariates and network tie-variables simultaneously. There has been some recent development in imputing missing network data based on conditional model distributions^4^, Bayesian multiple imputations^5,6^. (See also review and method comparisons by Huisman and Krause^7^ and Krause and colleagues^8^). Albeit these recent advances in exponential random graph models moved forward in that direction, developing a statistical tool that accounts for missingness for MERGMs was beyond the scope of this paper.

In scoring, we prioritised specificity over sensitivity in coding neutral and undecided responses (i.e., neither agree nor disagree and don’t know) as negative (with the exception of one item within the Adherence to positive sexual health norms index). It is important to note that these responses did not necessarily reflect students’ disagreement and thus these question answers might have overestimated misconceptions amongst students. Instead of using Likert-type scales to measure the extent of students’ agreement or disagreement, another possibility would be to assess one’s ability in controlling others as well as one’s own behaviours in different sexual situations and interactions using a self-efficacy scale with proof of good interrelation among question items represented by a high A Cronbach^3^. In future work, using different scales might help reducing the ambiguity about how to treat neutral choices when dichotomising question responses.

**Table S5. Parameter estimates for school 4**

|  | School4 |  |  |
| --- | --- | --- | --- |
| Effects | Parameter | Stderr |  |
| *Structural Effects* |  |  |  |
| Density [ArcA] | **-2.671** | **0.714** | ***** |
| Reciprocity [ReciprocityA] | 1.217 | 0.793 |  |
| Popularity spread [AinSA] | **-2.128** | **0.686** | ***** |
| Activity spread [AoutSA] | 0.009 | 0.431 |  |
| Path closure [ATA-T] | **1.292** | **0.286** | ***** |
| Multiple 2-paths [A2PA-T] | -0.256 | 0.193 |  |
| *Actor-relation interaction effects* |  |  |  |
| Gender-Sender | -0.109 | 0.59 |  |
| Gender-Receiver | -1.538 | 1.319 |  |
| Gender-Interaction | **3.543** | **1.309** | ***** |
| PS-Sender | 1.419 | 2.91 |  |
| PS-Receiver | **-13.027** | **4.959** | ***** |
| Knowledge-Sender | 0.072 | 0.175 |  |
| Knowledge-Receiver | 0.545 | 0.288 |  |
| Knowledge-Difference | -0.044 | 0.164 |  |
| Norms-Sender | 0.284 | 0.19 |  |
| Norms-Receiver | -0.579 | 0.324 |  |
| Norms-Difference | 0.022 | 0.137 |  |
| Talking-Sender | 0.114 | 0.173 |  |
| Talking-Receiver | -0.174 | 0.266 |  |
| Talking-Difference | **-0.283** | **0.1** | ***** |
| PS Talking-Sender | 0.062 | 0.406 |  |
| PS Talking-Receiver | -0.678 | 0.692 |  |
| *Cross-level effects* |  |  |  |
| Indegree [In2StarAX] | **2.456** | **0.945** | ***** |
| Outdegree [Out2StarAX] | -0.19 | 0.514 |  |
| Cross-level arc [TXAXarc] | **4.644** | **1.434** | ***** |
| Cross-level 3-paths [L3XAX] | **-0.583** | **0.18** | ***** |

In what follow, we present the GOF results and offer a brief explanation of it.

**Table S6. GOF results**

| Schools | 1 | 2 | 3 | 4 | 5 | 6 |
| --- | --- | --- | --- | --- | --- | --- |
| Statistics | t-ratio | t-ratio | t-ratio | t-ratio | t-ratio | t-ratio |
| In2StarA | 0.261 | -0.168 | 0.357 | -0.233 | -0.052 | 0.194 |
| Out2StarA | 0.285 | 0.137 | 0.142 | 0.15 | 0.176 | 0.237 |
| In3StarA | 0.66 | -0.416 | 0.977 | -0.55 | -0.173 | 0.314 |
| Out3StarA | 0.684 | 0.334 | 0.429 | 0.563 | 0.418 | 0.546 |
| TwoPathA | -0.399 | 0.041 | -0.42 | -0.118 | -0.2 | -0.104 |
| Transitive-TriadA | -0.757 | -0.026 | -0.663 | -0.107 | -0.61 | -0.215 |
| Cyclic-TriadA | -1.917 | -0.948 | -1.523 | -0.069 | -1.727 | -1.086 |
| T1A | -2.813 | -1.469 | -2.664 | -0.447 | -2.203 | -2.255 |
| T2A | -2.67 | -1.363 | -2.397 | -0.301 | -2.132 | -1.988 |
| T3A | -2.353 | -1.203 | -2.056 | -0.223 | -1.985 | -1.601 |
| T4A | -2.177 | -0.954 | -1.91 | -0.068 | -1.687 | -1.243 |
| T5A | -1.768 | -0.533 | -1.365 | -0.29 | -1.322 | -1.125 |
| T6A | -1.569 | -0.706 | -1.022 | -0.28 | -1.376 | -1.117 |
| T7A | -1.065 | -0.459 | -0.735 | -0.212 | -0.863 | -0.417 |
| T8A | -0.902 | -0.174 | -0.379 | -0.061 | -0.651 | -0.386 |
| SinkA | 0.589 | -0.628 | 0.213 | -0.309 | -0.576 | -0.567 |
| SourceA | 1.16 | -0.726 | 0.135 | -0.325 | -0.846 | 0.161 |
| IsolateA | -0.505 | 1.413 | -0.571 | 0.387 | 0.611 | 0.732 |
| AinAoutSA | -0.58 | 0.118 | -0.141 | -0.084 | 0.224 | -0.068 |
| ATA-C | -1.243 | -0.815 | -1.002 | 0.006 | -1.172 | -0.606 |
| ATA-D | 0.343 | 0.226 | 0.005 | -0.114 | 0.038 | 0.073 |
| ATA-U | -0.028 | 0.031 | -0.231 | -0.133 | -0.09 | 0.023 |
| ATA-TD | 0.255 | 0.116 | 0.006 | -0.086 | 0.049 | 0.084 |
| ATA-TU | 0.071 | 0.022 | -0.112 | -0.095 | -0.015 | 0.059 |
| ATA-DU | 0.157 | 0.125 | -0.116 | -0.124 | -0.028 | 0.048 |
| ATA-TDU | 0.161 | 0.087 | -0.074 | -0.102 | 0.002 | 0.064 |
| A2PA-D | 0.424 | -0.049 | 0.211 | 0.156 | 0.254 | 0.075 |
| A2PA-U | 0.534 | -0.309 | 0.48 | -0.298 | 0.002 | -0.062 |
| A2PA-TD | 0.195 | 0.007 | 0.038 | 0.024 | 0.122 | 0.056 |
| A2PA-TU | 0.235 | -0.112 | 0.165 | -0.144 | 0.037 | 0.004 |
| A2PA-DU | 0.509 | -0.231 | 0.394 | -0.047 | 0.113 | -0.007 |
| A2PA-TDU | 0.292 | -0.103 | 0.18 | -0.055 | 0.087 | 0.019 |
| gender_male_ActivityReciprocityA | -1.512 | -0.524 | -1.253 | -0.167 | -0.17 | -0.432 |
| gender_male_InteractionReciprocityA | -1.675 | -0.514 | -1.29 | -0.07 | -0.159 | -0.762 |
| PS_InteractionA | -0.761 | -0.412 | 0.292 | -0.493 | -0.575 | -0.505 |
| PS_ActivityReciprocityA | -1.784 | -1.019 | 0.014 | -0.619 | -0.433 | -0.244 |
| PS_InteractionReciprocityA | -1.384 | -1.276 | -0.928 | -0.74 | -1.308 | -0.52 |
| PS_In2StarA | 0.631 | 0.025 | -0.133 | -0.34 | -0.159 | -0.331 |
| PS_Out2StarA | 0.336 | 0.425 | 0.181 | 0.08 | 0.362 | 0.023 |
| PS_Mixed2StarA | 0.06 | -0.216 | 0.256 | 0.155 | 0.373 | 0 |
| KnowVar_SumA | -0.114 | 0.01 | -0.078 | -0.057 | 0.117 | 0.118 |
| KnowVar_ProductA | -0.529 | 0.276 | -0.168 | -0.201 | 0.209 | 0.052 |
| KnowVar_In2StarA | 0.056 | -0.002 | 0.167 | -0.284 | 0.046 | -0.064 |
| KnowVar_Out2StarA | 0.178 | 0.067 | -0.061 | 0.08 | 0.233 | 0.216 |
| KnowVar_Mixed2StarA | -0.455 | 0.288 | -0.615 | -0.045 | 0.026 | -0.303 |
| AttVar_SumA | 0.091 | -0.043 | -0.077 | -0.057 | 0.065 | 0.091 |
| AttVar_ProductA | -0.016 | -0.018 | -0.094 | 0.027 | 0.217 | 0.128 |
| AttVar_In2StarA | 0.292 | -0.119 | 0.202 | -0.282 | -0.107 | -0.054 |
| AttVar_Out2StarA | 0.162 | 0.029 | 0.054 | 0.303 | 0.04 | 0.169 |
| AttVar_Mixed2StarA | -0.461 | -0.217 | -0.338 | 0.157 | -0.238 | -0.265 |
| TalkVar_SumA | -0.029 | 0.086 | -0.028 | -0.098 | -0.129 | 0.054 |
| TalkVar_ProductA | -0.413 | -0.12 | -0.016 | -0.306 | -0.186 | 0.305 |
| TalkVar_In2StarA | 0.314 | -0.406 | 0.118 | -0.236 | -0.057 | -0.546 |
| TalkVar_Out2StarA | 0.341 | 0.343 | 0.059 | 0.067 | -0.038 | -0.139 |
| TalkVar_Mixed2StarA | -0.131 | 0.137 | -0.154 | 0.189 | -0.113 | -0.67 |
| PSTalk_SumA | -0.165 | -0.103 | 0.019 | -0.069 | 0.051 | 0.026 |
| PSTalk_DifferenceA | 0.576 | 0.318 | -0.172 | 0.208 | 1.679 | 0.041 |
| PSTalk_ProductA | -0.8 | -0.677 | 0.461 | -0.199 | -1.528 | -0.232 |
| PSTalk_In2StarA | 0.949 | -0.02 | -0.047 | -0.352 | -0.448 | -0.146 |
| PSTalk_Out2StarA | 0.279 | 0.361 | 0.1 | -0.13 | 0.009 | -0.01 |
| PSTalk_Mixed2StarA | 0.271 | -0.436 | 0.302 | 0.159 | -0.092 | 0.176 |
| AXS1Ain | -0.077 | -0.08 | 0.053 | -0.047 | 0.644 | -0.408 |
| AXS1Aout | -0.024 | -0.348 | -0.032 | -0.021 | 0.187 | -0.025 |
| AAinS1X | 0.178 | 0.156 | 0.153 | -0.219 | 0.198 | -0.243 |
| AAoutS1X | 0.311 | 0.103 | 0.37 | -0.183 | 0.288 | -0.082 |
| TXAXreciprocity | -1.576 | -1.34 | -0.911 | -0.534 | -1.029 | -1.066 |
| ATXAXarc | 0.895 | 0.215 | -0.05 | 0.469 | 0.278 | -0.159 |
| ATXAXreciprocity | -1.157 | -1.01 | -0.898 | -0.19 | -0.605 | -1.096 |
| L3XAXreciprocity | -1.796 | -1.314 | -0.889 | -0.543 | -1.146 | -1.033 |
| stddev_indegreeA | 0.453 | -0.279 | 0.837 | -0.181 | -0.067 | 0.362 |
| skew_indegreeA | 1.531 | -0.875 | 2.066 | -0.931 | -0.257 | 0.506 |
| stddev_outdegreeA | 0.577 | 0.45 | 0.699 | 0.337 | 0.592 | 0.771 |
| skew_outdegreeA | 0.837 | 0.397 | 0.627 | 1.438 | 0.439 | 0.68 |
| clusteringA_tm | -0.786 | -0.035 | -0.676 | 0.091 | -0.77 | -0.215 |
| clusteringA_cm | -2.429 | -1.306 | -1.941 | 0.159 | -2.443 | -1.514 |
| clusteringA_ti | -1.246 | 0.128 | -1.174 | 0.251 | -0.835 | -0.469 |
| clusteringA_to | -1.033 | -0.067 | -0.901 | -0.212 | -0.957 | -0.441 |

The GOF presents a list of statistics for not only the fitted statistics with estimated parameters presented in the fitted model, but it also includes additional statistics that are not included in the model. GOF test statistics are t-ratios comparing simulated graph statistic distributions and the observed statistics, where t-ratios smaller than 2.0 in scale suggest the observed statistics are not extreme in comparison with the model distribution, hence adequate fit. T-ratios greater than +2.0 indicate that the model failed to produce as many configurations as in the observed network. If, in the opposite, we have t-ratios smaller than -2.0, it means that the model failed to produce as few configurations as the observed network. With MPNet we simulated 10,000,000 graphs with the fitted model and took every 1,000^th^ graph to form a sample of 10,000 graphs. Then, the software compared the results of the sample with the observed network.

References

1. Little, R. J. A. Missing-Data Adjustments in Large Surveys. *J. Bus. Econ. Stat.* **6**, 287–296 (1988).

2. Heitjan, D. F. & Little, R. J. A. Multiple Imputation for the Fatal Accident Reporting System. *J. R. Stat. Soc. Ser. C (Applied Stat.* **40**, 13–29 (1991).

3. Rostosky, S. S., Dekhtyar, O., Cupp, P. K. & Anderman, E. M. Sexual self-concept and sexual self-efficacy in adolescents: a possible clue to promoting sexual health? *J. Sex Res.* **45**, 277–286 (2008).

4. Wang, C., Butts, C. T., Hipp, J. R., Jose, R. & Lakon, C. M. Multiple imputation for missing edge data: A predictive evaluation method with application to Add Health. *Soc. Networks* **45**, 89–98 (2016).

5. Koskinen, J. H., Robins, G. L. & Pattison, P. E. Analysing exponential random graph (p-star) models with missing data using Bayesian data augmentation. *Stat. Methodol.* **7**, 366–384 (2010).

6. Koskinen, J. H., Robins, G. L., Wang, P. & Pattison, P. E. Bayesian analysis for partially observed network data, missing ties, attributes and actors. *Soc. Networks* **35**, 514–527 (2013).

7. Huisman, M. & Krause, R. W. Imputation of Missing Network Data. in *Encyclopedia of Social Network Analysis and Mining* (eds. Alhajj, R. & Rokne, J.) 1044–1053 (Springer New York, 2018).

8. Krause, R. W., Huisman, M., Steglich, C. & Snijders, T. Missing data in cross-sectional networks – An extensive comparison of missing data treatment methods. *Soc. Networks* **62**, 99–112 (2020).
